# A feminine advantage in the domain of harm: a review and path forward

**DOI:** 10.1098/rsbl.2024.0381

**Published:** 2024-11-13

**Authors:** Maja Graso, Tania Reynolds

**Affiliations:** ^1^ Psychology, University of Groningen Faculty of Behavioural and Social Sciences, Groningen, The Netherlands; ^2^ University of New Mexico College of Arts and Sciences, Albuquerque, NM, USA

**Keywords:** biases, harm perception, morality, gender

## Abstract

Despite well-documented disparities disadvantaging women (e.g. discrepancies between men and women in salaries and leadership roles), we argue that there are contexts in which disparities disadvantage men. We review the literature suggesting harm to women is perceived as more severe and unacceptable than identical harm to men, a bias potentially rooted in evolutionary, base rate, stereotype-based and cultural shift explanations. We explore how these biases manifest in protective responses toward women and harsher judgements toward men, particularly in contexts of victimization and perpetration. Our review aims to complement the existing literature on gender biases by presenting a balanced view that acknowledges men and women face unique challenges. By understanding these biases, we hope to foster a more equitable discourse on gender and harm, encouraging empathy and validation of suffering irrespective of gender. This holistic approach aims to de-escalate gender-based conflicts and promote effective interventions for both men and women.

## 1. Introduction

Disparities between men and women persist, with women often experiencing poorer outcomes. News outlets frequently document women’s diminished salaries and under-representation in positions of decision-making power [1,2], academics study the sources of those discrepancies (e.g. [3,4]) and practitioners often implement various interventions and mentorship pathways to aid women in gaining equal access to coveted resources [5]. Among numerous identified roots of those challenges, ranging from boys’ clubs [6] to the motherhood penalty [7], biases emerge as among the most frequently cited and studied explanations.

Supporting the contributing role of biases in perpetuating female disadvantage, some experimental research reveals that otherwise identical behaviours or contributions are evaluated differently based on the gender of the person associated with them. For example, identical artwork is rated as more valuable when it is ostensibly created by an artist with a stereotypical man’s name versus a woman’s name [8]. Similarly, identical curricula vitae are evaluated as higher quality and with greater leadership potential when they belong to a male versus female scientist [9,10]. To be sure, however, more recent research is beginning to question the prevalence and extent of biases against women by highlighting areas where women are on par with men or actively preferred (e.g. [11–13]). Nonetheless, certain disparities disfavouring women persist, such as among teaching ratings and salaries [11], which tend to garner attention and carry weight in public narratives.

We maintain that just as biases and stereotypes can disadvantage women (often to the benefit of men), there might be contexts in which similar biases and stereotypes disadvantage men (and perhaps benefit women). We contend that one major domain where women are advantaged relative to men is soliciting observers’ concern over their suffering. Consider the historical expression ‘women and children first’ (*Birkenhead* drill), which underscores the priority given to women in situations of harm [14]. Although this phrase might now seem dated and no longer evoke benevolent intentions, similar sentiments still persist, as across numerous contexts, harm to women is perceived as more severe, troubling and unacceptable than identical harm befalling men [15]. Consequently, people may be more wary of placing women in harm’s way than men [16].

Our review has several objectives. First, although gender bias in harm-based contexts has been documented, it tends to be absent from the widely cited reviews of gender biases [17,18]. Therefore, with the hope of complementing the reviews emphasizing biases disadvantaging women, we openly consider and document evidence that some biases may, in fact, disadvantage men. Second, we invite researchers to consider the merits of de-escalating tensions between men and women by acknowledging that they face their respective challenges, perhaps partly because biases can be simultaneously advantageous and disadvantageous, dependent on the context. Last, we engage with the empirical literature documenting asymmetries in harm perceptions, aiming to compile a more holistic view and informed understanding of prevailing gender biases and their implications. By understanding these biases and their nuanced effects, we hope to contribute to a more equitable and informed discourse on gender and harm.

We begin our review by first examining four potential sources of gender partiality in harm perceptions. These include *evolutionary origins*, *base rates*, *gender-biased moral typecasting* and *cultural movements*. Second, we review evidence for gender asymmetries in harm detection across the domains of victimization and perpetration. Last, we discuss the implications of these findings for contemporary discourse and identify key questions that remain understudied.

## 2. Sources of gender-based partiality

Several perspectives offer insights into why people might be partial when evaluating harm to men and women. Although the following explanations are certainly not exhaustive, this extant literature nonetheless provides a starting point to inform fruitful pathways forward.

### (a) Evolutionary origins of prioritizing harm to women: demands of childcare

From an evolutionary standpoint, communities might seek to preferentially insulate women from certain kinds of harm due to their central role in reproduction. Women’s bodies are responsible for internal gestation and lactation. Due to these substantial physiological contributions, which are costly in terms of both calories and time, women set the upper limit on reproduction [19]. Consider a group of 20 men and only four women. This group could produce far fewer offspring than one with the reverse ratio: 20 women and only four men. This asymmetry in reproductive value might have favoured social institutions that protect women against physical threats [20]. That is, cultural evolution might have favoured groups that contained norms or institutions prioritizing female safety. Such groups would have produced more offspring, expanded more rapidly, and enjoyed numerical advantages on battlefields compared to groups with no such institutions or institutions that prioritized male safety [21]. Similarly, individuals might have preferentially migrated to groups with such protective norms upon observing that these groups successfully insulated their female members against harm [22].

Following childbirth, women contribute the preponderance of childcare around the globe [23,24]. Thus, not only do women make substantial physiological investments in reproduction, they also allocate the majority of care, protection and instruction infants and young children receive. Moreover, human children require care, attention, and resources far longer than almost all other mammals [25,26]. If children require substantial resources from their mothers to reach sexual maturity—and if social groups cannot propagate without ensuring individuals reach maturity to reproduce—it stands to reason that groups who protected children and, by extension, those who are most closely responsible for their upbringing, would be those that spread most effectively.

On average, then, modern human groups should possess cultural norms or social institutions that allocate aid and protection to women. Even today, despite modern women bearing fewer children than ancestral women and expanding their roles to numerous tasks beyond childcare (e.g. greater participation in the labour force), child-rearing nonetheless remains a costly endeavour physically [27], psychologically [28] and professionally [7]. Such protective norms could manifest as perceptions that women’s suffering is more upsetting and less acceptable than men’s because harm to women is more detrimental to societal survival [19,23].

### (b) Base rates origins of prioritizing harm to women: disproportional capacity for aggression and harm

Beyond evolutionary logic, differences in men’s and women’s capacity and likelihood to inflict or experience harm may shape gendered expectations. In short, gendered base rates of enacting or experiencing harm drive social assumptions about likely perpetrators and victims ([29,30]). If we consider base rates of harm infliction, men commit the majority of physical violence, a trend that is consistent across cultures [31–33]. Across history, men were nearly the sole participants in war [16,23,34]. Thus, out-group men often raided ancestral groups, leading to heightened perceptions of men as sources of danger. Moreover, not only are men more often the perpetrators of violent aggression, but their bodies also appear to be shaped specifically for success in violent combat. They have 61% more musculature than women [35], which is particularly concentrated in the upper body [36], leading them to be substantially stronger [37]. Compared to women, men also show advantages in handgrip strength [38] and throwing ability [39]. Thus, not only do men enact more physical harm onto others, but they are also more capable of inflicting substantial harm than women.

Just as base rate differences in propensity and capacity to enact harm might influence social expectations of perpetration, average differences between men and women might also shape assumptions about vulnerability. When interactions between men and women become physically violent, women are especially vulnerable to experiencing bodily harm. Although men are more often the victims of physical violence from other men, as the majority of physical violence is man-on-man [31,40], when men perpetrate violence against women, the situations are markedly uneven. Specifically, women are generally at a disadvantage relative to men in terms of size [41] and strength [38]. These physical differences skew the potential harm of physical conflicts, especially in cases of intimate partner-perpetrated physical violence [32,33,42].

Additionally, women face significant risks from sexual violence, such as rape, which not only causes severe physical and psychological trauma [43–45], but also impinges on their reproductive autonomy by denying them the choice of partners who might otherwise have provided support, investment or desired genetic attributes. Although men certainly do experience and suffer from sexual violence [46], these experiences less often threaten men’s reproductive autonomy compared to when women suffer this violence. That is, female sexual assault victims could be forced to gestate, birth and raise resultant children, circumventing their autonomy to select mates or decide to reproduce altogether. From an evolutionary standpoint, women’s particular vulnerabilities to physical and sexual aggression might contribute to why societies exhibit heightened sensitivity and protective norms toward women compared to men [32,33].

Gender differences in self-perceptions, values and experiences might further contribute to expectations about which individuals enact versus experience harm. Men generally rate themselves higher in agency and lower in communion compared with women, suggesting self-views that align with assertiveness and greater inclinations to enact harm [47]. Additionally, across cultures, men prioritize power and self-direction more strongly, whereas women place higher importance on universalism, benevolence and safety [48]. Although these divergences in values might not always be immediately observable, they likely contribute to perceptible gender differences in behaviors and choices. In line with expectations of women as vulnerable to harm, women report experiencing greater stress than men in their daily lives [49]. Moreover, compared with men, women report worse physical and mental health [50] and more often seek medical assistance [51,52]. To be sure, however, men die at earlier ages than do women [38]. Thus, although objective metrics of suffering might not support women’s greater vulnerability, women might nonetheless experience the same ailments as subjectively more distressing than men and more readily communicate this suffering to others.

These differences in perceptions and values might have evolutionary underpinnings. Campbell [53] argued that because mothers more strongly contribute to offspring survival than fathers, women should generally avoid physical aggression. Benenson *et al*. [54] expanded this theorizing to contend that women have evolved numerous strategies to keep themselves alive, such as heightened biological and psychological sensitivity to threats (e.g. stronger immune responses, greater fear). That is, if women play a more critical role in children’s survival, women should possess a wide array of adaptations to keep themselves safe. For instance, women tend to be much more risk-averse than men and espouse broader definitions of harm. This risk aversion manifests in contexts ranging from financial decisions and high-risk ventures to endorsing social policies that protect against the risks of poverty [55–59]. Thus, if women evolved a heightened sensitivity and motivation to avoid harm compared to men, individuals might detect these differences and, consequently, be more attuned to women’s suffering [54].

### (c) Moral typecasting: why it is easier to envision men as perpetrators of harm

If people have faced cultural incentives to protect women from harm or are merely recognizing gender-differentiated base rates of harm infliction/vulnerability, then they might possess cognitive shortcuts attuned to such differences. That is, individuals might evince perceptions that cast women into roles needing protection and men into roles of harm perpetration. Moral typecasting theory contends that when humans observe instances of harm, they instinctively cast the involved parties into the agentic roles of the harm-inflicting perpetrator or the passive role of the suffering victim [60]. This dyadic template generates expectations of an intentional perpetrator and a suffering victim, with anger directed at perpetrators and compassion toward victims. Importantly, these roles are mutually exclusive, making it difficult to perceive someone as both ([60]). Likewise, once a target is recognized as a victim, they are less likely to be recognized as a perpetrator, and *vice versa*. This cognitive heuristic is powerful enough to ‘complete the dyad’, such that individuals imagine or generate perpetrators when harm is detected.

Given the evolutionary logic and base rate differences outlined above, gender stereotypes could significantly influence the ease with which men and women are perceived as fitting the roles of perpetrators or victims. Men tend to be associated with traits denoting power and status [61,62], and they tend to be considered less warm than women [63]. These traits all connote agency or the ability to exert one’s will in the world, which should facilitate classification into the agentic role of perpetrator. Indeed, people hold implicit associations with masculinity and the potential for harm, such as rage-driven violence [64]. Furthermore, because the roles of perpetrator and victim are mutually exclusive and inversely related (60), then greater ease in classifying men as perpetrators should correspond to greater difficulty in recognizing their suffering or classifying them as victims.

Just as associations of men with stereotypes denoting agency may benefit them in contexts in which agency is highly valued, such as in positions of power [65,66], associations of women with passivity or warmth might similarly advantage them in certain contexts. Women tend to be associated with stereotypes pertaining to interpersonal warmth or communion [17,67,68]. Specifically, Eagly & Mladinic described this gendered bifurcation as the ‘women are wonderful’ effect, whereby women are often evaluated more favourably due to their presumed commonality, whereas men are viewed less favourably due to their presumed assertiveness and lower warmth [69,70]. Across cultures, women were perceived as less powerful than men but were seen more positively [61]. High warmth and low agency are traits that tend to evoke pity [71], suggesting that these female stereotypes should increase the likelihood women are typecast into the pity-evoking role of victim.

Experimental investigations support that women are more readily typecast as victims and men as perpetrators [72]. For example, when participants were exposed to scenarios depicting harm, they more often recalled that victims were female than male, even though the victims’ gender was left unspecified. In another study that used videos of animated triangles, Reynolds *et al*. [72] found that the more individuals perceived a triangle as a victim of interaction, the more likely they were to classify that triangle as female, whereas the more individuals perceived a triangle as a perpetrator, the more likely they were to classify it as male. These findings suggest that people exhibit a gender bias in moral typecasting, such that they more readily identify men as perpetrators and women as victims.

### (d) Cultural trends over time

Although the arguments mentioned above likely explain some portion of the greater concern over female suffering, they cannot account for change over time or variability across cultures. Indeed, in most countries, women show more favourable outcomes than men (as measured by life satisfaction, healthy life span and educational enrolment; [73]). However, this is not true across all countries, and such female advantages are associated with greater levels of human development [73]. Thus, development appears to be associated with enhanced outcomes among women. One explanation for development’s enhancing treatment of women is that as threats to survival are minimized, individuals and societies more strongly prioritize self-expression values, which include support for gender equality [74]. That is, when people experience existential security, concerns over safety and survival become supplanted by other values, including tolerance and freedom. Thus, as the world becomes increasingly safe, individuals begin to prioritize goals related to well-being over those related to mere survival.

Beyond safety-driven shifts towards prioritizing equality and well-being, active efforts from women’s movements also likely catalysed concern over issues afflicting women. At the end of the nineteenth century, the first international women’s organizations were formed, championing women’s right to vote and participate in education and labour forces [75,76]. These institutions integrated women’s perspectives into global policy-making [77]. Levering justice-based arguments for equal access to leadership and protectionist arguments against the threats of sexual harassment and hostility, feminist movements succeeded even among conservative institutions such as churches and militaries [78]. Indeed, support for feminism and gender equality has continued to rise over time [79], with younger individuals continually showing more favourable attitudes than older generations [80,81].

Beyond younger age, one of the strongest predictors of feminist identification and egalitarian gender attitudes is heightened education [80,82]. Longitudinal analyses support that students become more egalitarian about gender issues during college [83]. Thus, academia might be an especially liberalizing environment. Indeed, American estimates suggest the majority of faculty identify as liberals [84], a pattern that is especially pronounced in the social sciences [85].

Such a left-leaning environment might have proved especially conducive to feminist perspectives. Indeed, evidence suggests a pro-female bias across various stages of academia. For example, the percentage of women enrolling in STEM graduate programmes and receiving PhDs has increased since the 1970s, despite no changes in women’s lower GRE scores relative to men’s [86,87]. Similarly, the percentage of female tenure-track assistant professors has increased over time [87]. Controlled experiments reveal strong hiring biases in favour of female applicants for both assistant and associate professor positions [13,88]. Likewise, women are promoted to full professors with fewer publications and citations than men promoted to the same positions [89,90].

Because women tend to hold more positive attitudes toward feminism and egalitarian gender relations [79,80,83,91], these trends are likely to be somewhat self-perpetuating. That is, the pro-female bias in academia should lead to increasing proportions of female students and faculty, who, on average, might favour feminist arguments, further propelling the pro-female bias. Thus, although there are secular trends toward increased acceptance of feminism, perhaps as a result of greater safety, these narratives might be especially welcomed in contexts that have higher percentages of liberal-leaning individuals or women, such as academia.

## 3. Evidence of partiality

We have identified evolutionary pressures, base rates, stereotypes, and cultural shifts as four potential explanations underlying gender-based partiality in judgements of harm. Next, we review the evidence that people exhibit systematic biases in the two primary domains of harm appraisals: victimization and perpetration. We prioritize experimental investigations, as these can hold the suffering constant, uncovering true biases in moral responses.

### (a) Partiality in detecting and responding to victimization

Our four proposed explanations—or some combination of them—should lead to the prediction that people will exhibit particularly protective and sympathetic responses to women’s suffering relative to men’s. Evidence for such a bias in harm detection manifests in contexts where harm is severe (e.g. death) to less tangible forms, including symbolic depictions. Sacrificial harm refers to scenarios where individuals must be harmed or sacrificed for the greater good [92–94]. In these situations, people find it more acceptable to sacrifice men than women. For instance, using hypothetical trolley dilemmas, participants were less willing to push a woman onto the tracks to her death than a man [95], a finding that was also replicated in a virtual reality depiction of the same dilemma [96]. These patterns suggest that when life or death is at stake, people are more willing to save a woman’s life. Indeed, such a pattern would make sense if women contribute more to reproduction, and thus, preserving women’s lives over men’s would preserve reproductive output. In line with this logic, one study found that the bias to save a woman’s life over a man’s disappears when targets are older than middle-aged [19]. That is, women are not preferentially saved when they can no longer reproduce.

This bias to protect women extends beyond sacrificial dilemmas and manifests in lower-level physical and psychological harm. When individuals were given the opportunity to gain money at another’s expense, they sacrificed more money to prevent a woman from experiencing painful shocks than a man [95]. People also reported experiencing more pity when exposed to photos of women (versus men) in pain [97]. This gender bias was again eliminated when the individuals were outside of their reproductive windows (e.g. elderly), further suggesting that the greater tendency to protect women is rooted in asymmetries in reproductive capacities. In a study of interpersonal harm, people felt more sympathy for and were less dismissive toward women disclosing poor treatment by same-gender friends compared to men sharing identical disclosures of poor treatment [98]. In another set of experiments, Graso *et al*. [99] found that people were less willing to support interventions that caused collateral harm to women than those that caused identical harm to men. For example, participants were less supportive of an intervention programme offending women than one offending men, even if it benefitted many people. They were also less willing to endorse a weight loss drug that improved overall well-being but had side effects for women, compared with a drug similarly affecting men. Even in traditional contexts where women are expected to sacrifice, such as caring for infants and the elderly, people were more likely to endorse interventions that inflicted incidental harm onto men than onto women.

This greater concern over harm to women also manifests at the collective level in response to group-based asymmetries. One experiment found that when women were fired, people assumed they experienced more pain and that their harm was more intentional than when men were fired [72]. Such disparities extend to real-world asymmetries. People more strongly believe women’s under-representation in male-dominated fields (e.g., engineering) reflects societal discrimination and warrants social action compared with men’s under-representation in female-dominated fields (e.g. nursing [100]). Moreover, studies of forecasting accuracy reveal that people overestimate the degree of hiring discrimination against women and underestimate the degree of discrimination against men [12]. These data imply that people more readily detect harm to women, perhaps because they more closely fit the victim prototype than do men.

This greater concern over harm to women extends to symbolic forms. Using experimentally manipulated popular science articles, Stewart-Williams *et al*. [101,102] demonstrated that people evaluate identical scientific findings as more harmful, less important, and less credible when they reveal women score lower in a socially valued domain (i.e., drawing) or higher in a socially condemned domain (i.e., frequency of lying) compared to men. These findings suggest an aversion to even symbolic forms of harm, whereby scientific truths portray women in an unflattering light.

### (b) Partiality in detecting and responding to perpetration

If men, due to their greater capacity for and inclination toward physical violence, more closely match the cognitive prototype of perpetrators, it should be easier to detect when they have inflicted harm and experience the corresponding moral responses, such as condemnation. These predictions are borne out in experimental paradigms that examine sexual harassment. Experiments reveal people perceive the same actions as more sexist, harassing, and insulting when they are committed by a man than a woman [103,104]. Such perceptions extend to moral responses, such that people desire harsher punishments and are less willing to forgive a man (versus a woman) who makes a potentially inappropriate comment at the workplace [72].

These trends indicate that men are more harshly evaluated than women, which is mirrored within hypothetical and real-world courtrooms. Numerous investigations reveal that among mock jurors, male (versus female) defendants are treated more harshly [105–108]. These biases manifest in real-world judicial decisions, such that male defendants are more likely to be convicted and receive longer sentences than female defendants, even after accounting for the severity of the crime and the defendants’ criminal history [109–114]. These well-replicated findings suggest that perhaps because men more closely fit the prototype of perpetrators, it is cognitively easier to detect their infliction of harm, leading to harsher and more punitive responses.

## 4. General discussion

We have reviewed evidence that people are less sensitive to harm befalling men compared to identical harm befalling women. Our examples span various types of harm, including sacrificial or instrumental harm enacted for some common good, general suffering and responses to allegations of wrongdoing. Across these cases, research indicates partiality that tends to benefit women and disadvantage men. We have traced this partiality to several factors ranging from evolutionary disparities to stereotypes. However, this review represents only a snapshot of broader patterns of harm experienced by men and women. We do not claim that either men or women suffer more than the other, nor do we negate the very real differences in experiences between men and women. This review is not definitive, but is an opportunity to consider the current research on biases, identify potential implications, and invite additional research. We hope that by doing so, such nuance can de-escalate gender-based conflicts and promote empathy and validation of suffering, irrespective of gender.

### (a) Implications for research

Although many scholars, journalists and social commentators have lamented the under-recognition of female agency and competence, few have acknowledged the potential benefits of such stereotypes for women (and potential harm for men). If it is cognitively more challenging to recognize men as victims, perhaps due to increased perceptions of agency, then society might under-acknowledge or under-address areas where men suffer. For example, compared to women, men are more likely to be homeless [115], drop out of school [116], suffer from substance abuse [117] and die by suicide [118]. These trends suggest there is widespread suffering among men. If individuals tend to show diminished concern in response to the suffering of men (versus women), then it is possible that the sources, contributing factors, and potential interventions of this suffering could be under-investigated, under-funded or under-acknowledged. Perhaps by shedding light on this diminished concern, empirical investigations can stimulate further investigation, funding and policies to more effectively address male suffering.

Just as the biased application of moral typecasting might lead to an under-recognition of male suffering, it might also lead to an under-detection of female perpetration [72,119]. As noted above, female defendants are less likely to be convicted than male defendants. Thus, legal institutions might fail to protect potential future victims from dangerous perpetrators. Indeed, females commit about 20% of violent crimes [120]. Moreover, female perpetrators are more likely to target female than male victims [120]. Thus, if society is especially concerned about preventing female harm, understanding female perpetration might be one critical step. However, it might be especially challenging to recognize and respond to harm when neither the victim nor the perpetrator matches gender prototypes. For instance, male victims of female-perpetrated intimate partner violence are met with ridicule, doubt and minimization [121], despite evidence suggesting women commit intimate partner violence at similar rates to men [122].

In addition to overlooking female perpetrators, it is possible that male-biased assumptions of harm might fail to detect patterns of aggression that are female-typical. Compared to men, women are equally or more likely to engage in indirect aggression, which employs tactics such as spreading rumours, ostracism, dirty looks and veiled gossip [40,123–125]. This form of aggression often aims to harm the social standing of a victim while simultaneously avoiding detection as the aggressor. If the cognitive prototype of a perpetrator is masculine, then future investigations might profit from examining whether the cognitive prototype of harm is also male-typical and, thus, whether individuals fail to detect more feminine manifestations of harm, such as reputational smears online or emotional manipulation.

By examining the numerous manifestations of gender bias, empirical research might inform and temper societal discussions of fairness. The same gender stereotypes that link men with agency and women with passivity might advantage men in boardrooms, offices or voting booths, but disadvantage men in hospitals, homeless shelters, charity campaigns or courtrooms. Thus, it remains unclear whether either sex is at an overall disadvantage. Rather, each suffers uniquely in differing contexts, dependent upon whether agency is advantageous. Perhaps by presenting a more even-handed depiction of gender prejudice and discrimination, individuals can feel both more recognized for their suffering and also more empathetic for the suffering of other genders. Online communities, such as those who identify as involuntarily celibate [126], suggest gender relations might be tenuous or deteriorating. Better understanding the sources of grievances and resentments might be an important step in promoting compassion between men and women.

### (b) Under-examined questions and future directions

#### (i) What is the effect of culture on gender partiality in harm perceptions?

The studies we reviewed were confined primarily to Western samples. There is certainly ample research documenting gender disparities beyond just the WIERD cultures [127], but studies based on experimental work are lacking. Cultural contexts can profoundly influence how harm is perceived and evaluated, and gender roles might vary greatly from one society to another [128], thereby potentially altering harm evaluations. For instance, it is possible that in cultures where traditional masculinity is highly valued and associated with resilience and strength, sacrificial harm to men might be perceived as especially admirable or as the fulfilment of traditional roles. Conversely, in contexts where femininity is strongly linked to expectations of reproduction and heavy investment in childcare, harm to women—even if sacrificial and for the greater good—might be viewed as more objectionable or alarming. If cultural factors can shift the relative acceptability of sacrificial harm to men versus women, cross-cultural research is needed to better understand this variability and its causes.

#### (ii) How do trait differences influence harm perceptions?

Our analysis has predominantly focused on the binary categories of men and women. This focus was due to the preponderance of research examining differences in how harm is perceived when inflicted upon men versus women. However, we acknowledge that treating gender identity and sex as synonymous might lead to oversimplified conclusions or reinforce traditional binary gender norms, limiting our understanding of harm perceptions across a wider spectrum of individuals. Future research could benefit from examining whether the average characteristics differentially associated with men and women (e.g. muscularity, emotional sensitivity) influence harm perceptions above and beyond the person’s biological sex.

Such investigations might shed light on how individuals assess harm to those possessing traits that violate gendered expectations. For instance, traits typically associated with masculinity (or agency) might predispose individuals to be perceived as perpetrators, regardless of their biological sex or gender identity, while traits aligned with femininity (or passivity) might increase the likelihood those individuals are perceived as victims [97,129]. Alternatively, it is plausible that men who display highly feminine traits might receive diminished sympathy, as their defiance of gender expectations of masculinity can evoke social punishment (for the backlash in stereotype maintenance, see [130]). Whether these backlash effects occur in the context of harm warrants investigation. It is possible that although masculine women or feminine men generally experience social penalties, the degree to which they evoke sympathy or blame in the context of harm is predicted by their cues of relative patiency or agency. Such explorations are timely and necessary, as they could reveal underlying biases in harm perception that are influenced by gendered associations and expectations rather than by biological sex *per se*. Furthermore, as gender norms (and stereotypes) vary and evolve [67], there is also a need to assess continually how these dynamics correspond to recognition of and responses to harm.

#### (iii) Do female-favouring gender biases manifest outside harm?

This review investigated whether biases and disparities favouring women manifest in the context of harm. Indeed, sex differences in contributions to reproduction provide a rationale for a bias to protect women from harm, just as base rates might justify an inclination to perceive men as perpetrators. However, it is also possible that biases favouring females manifest in other contexts, such as in responses to positive actions (e.g. praise) or receiving positive treatment (e.g. perceptions of deservedness). Indeed, Seager & Barry [119] contend that pro-female biases exist across all four dimensions: negative action (blame), negative treatment (sympathy), positive action (praise) and positive treatment (perceptions of deservingness). The current review forwarded evidence in support of the first two categories. However, future research would benefit from examining the latter two domains to elucidate whether women receive more praise for their accomplishments or good deeds than do men and whether women’s positive treatment is evaluated as fairer or more deserved than men’s.

## 5. Conclusion

This review aimed to complement, rather than supersede, the existing literature on the nature and impact of biases against women. Just as it is crucial to examine stereotypes and prejudice that might disadvantage women, it is also valuable to document and reflect on empirical evidence suggesting deleterious assumptions and treatment of men. Such considerations might highlight contexts of overlooked suffering, opportunities for intervention, or sources of miscommunication between men and women. By providing a more balanced and even-handed assessment of gender disparities in assumptions and societal treatment, researchers might identify domains of future exploration, policymakers might correct oversights, and individuals might develop compassion for alternative forms of suffering.

## Data Availability

This article has no additional data.
